# Composition of the infiltrating immune cells in the brain of healthy individuals: effect of aging

**DOI:** 10.1186/s12979-022-00302-y

**Published:** 2022-10-08

**Authors:** Tapio Nevalainen, Arttu Autio, Mikko Hurme

**Affiliations:** 1 Faculty of Medicine and Health Technology, Tampere University, Arvo Ylpön katu 34, 33520 Tampere, Finland; 2 Gerontology Research Center (GEREC), Tampere, Finland

**Keywords:** Brain, Infiltrating immune cells, Aging, Inflammaging

## Abstract

Immune cells infiltrating the central nervous system (CNS) are involved in the defense against invading microbes as well as in the pathogenesis of neuroinflammatory diseases. In these conditions, the presence of several types of immune and inflammatory cells have been demonstrated. However, some studies have also reported low amounts of immune cells that have been detected in the CNS of healthy individuals, but the cell types present have not been systematically analyzed. To do this, we now used brain samples from The Genotype- Tissue Expression (GTEx) project to analyze the relative abundance of 22 infiltrating leukocyte types using a digital cytometry tool (CIBERSORTx). To characterize cell proportions in different parts of the CNS, samples from 13 different anatomic brain regions were used. The data obtained demonstrated that several leukocyte types were present in the CNS. Six leukocyte types (CD4 memory resting T cells, M0 macrophages, plasma cells, CD8 T cells, CD4 memory activated T cells, and monocytes) were present with a proportion higher than 0.05, i.e. 5%. These six cell types were present in most brain regions with only insignificant variation. A consistent association with age was seen with monocytes, CD8 T cells, and follicular helper T cells. Taken together, these data show that several infiltrating immune cell types are present in the non-diseased CNS tissue and that the proportions of infiltrating cells are affected by age in a manner that is consistent with literature on immunosenecence and inflammaging.

## Introduction

The central nervous system (CNS) was originally regarded as a site with “immune privilege”, i.e. being outside of the extensive immune surveillance mechanisms. However, it is now known that CNS contains distinctive immune mechanisms, which are involved both in the defense against invading microbes as well as in the various physiologic maintenance mechanisms in this organ. Two different mechanisms have been characterized differing in the cell types involved. The first is based on microglia and astrocytes, while the second is caused by peripheral immune cells that infiltrate the CNS.

The infiltration of immune cells from the blood to the CNS is effectively regulated by the blood-brain barrier (BBB). It is now known that breakdown of the BBB is a crucial step in the pathogenesis of several infectious and inflammatory diseases in the CNS [1] and the associations between the presence of a certain immune cells and disease susceptibility/severity have been observed. For example, in multiple sclerosis (MS), a prototype autoimmune disease of the CNS, both tissue-resident CD8+ T cells and B cells can be found in the CNS [2, 3]. These tissue-resident CD8+ T cells are probably also induced in several viral infections in the CNS [4]. In Alzheimer’s disease the situation appears to be more complicated: Liu et al. [5] observed several immune cell types (both of the innate and adaptive cell types) in the CNS with variable levels probably reflecting the complexity of the pathogenesis of this disease.

The proportions of immune cells present in the blood are well established, but their numbers as infiltrated cells in the CNS are not well characterized, and neither are the mechanisms of the infiltration. Infiltrating immune cells are known to be present in the CNS of even healthy individuals, at least in low quantities and at least some of the time. In the case of active infection or autoimmune reactions, the breakdown of the BBB is probably the decisive mechanism allowing the infiltration to the CNS. In addition to the BBB, there are two other barriers, the blood-leptomeningeal barrier (BLMB) and the blood-CSF barrier (BCSFB) and it has been suggested that only BCSFB allows the transmigration to the CNS in healthy individuals [6].

To be able to understand the immune or pathogenetic mechanism of the immune cells found in the CNS, it would be important to know their anatomic location as well as the effects of the various intra-CNS mechanisms to their amount and activity. To comprehensively characterize the CNS infiltrating immune cells in healthy individuals we now determined the abundance profile of 22 leukocyte types from brain samples using a digital cytometry tool (CIBERSORTx) [7]. The brain samples originate from 13 different brain anatomic regions, which were also compared in analysis.

Another factor that has wide impact on the function of the immune system, including on cell type proportions, is age [8]. Age-associated changes to the function of the immune system are commonly referred to as immunosenescence. Age-associated changes to cell type proportions in the blood are well characterized, and such age-associated changes might be seen in leukocyte infiltration to the CNS as well.

## Materials and methods

### Origin of raw data

The polyA-enriched RNA-sequencing data studied in this work originates from non-diseased region-specific brain samples taken as part of the Genotype-Tissue Expression (GTEx) Project (dbGaP accession number phs000424.v8.p2) [9]. The GTEx project as a whole is an ongoing effort to build a comprehensive public resource to study tissue-specific gene expression and regulation. As part of the project, 17,382 samples have been collected from organ and tissue donors, originating from 54 types of tissue and from 948 individuals. Samples used in the project are collected from non-diseased tissue sites and are studied using primarily molecular assays, including WGS, WES, and RNA-Seq.

### Donors and samples

The 378 region-specific brain samples studied in this work originate from 55 persons. Ages of the sample donors varied between 21 and 70 years, with the mean age being 56.9 years (Std deviation 11.1). Thirty-seven samples were from male and 18 from female donors (male mean age 56.1 years, female mean age 58.14 years). All GTEx sample donors were surgical patients or post-mortem donors [9]. Eligibility criteria and sequencing of the biological samples has been described in more detail elsewhere [9, 10]. The samples originated from 13 brain regions: amygdala (*n* = 24), anterior cingulate cortex (*n* = 24), caudate (*n* = 35), cerebellar hemisphere (*n* = 37), cerebellum (*n* = 39), cortex (*n* = 31), frontal cortex (*n* = 31), hippocampus (*n* = 28), hypothalamus (*n* = 25), nucleus accumbens (*n* = 34), putamen (*n* = 30), spinal cord (*n* = 20) and substantia nigra (*n* = 20). The average number of samples from a single individual was 6.9 (Std deviation 3.8, min 1, max 13).

### Pipelines

A bioinformatics pipeline was run in Puhti supercomputer cluster of CSC (Espoo, Finland) to quantify gene expression in the samples from RNA sequencing data. Paired-end RNA-seq reads of 378 brain samples were downloaded from Sequence Read Archive in FASTQ format with SRA Toolkit (v2.10.8). Low-quality ends (Phred score < 20) and Illumina Universal Adapters were trimmed with TrimGalore (v0.6.4; https://github.com/FelixKrueger/TrimGalore; 10.5.2021). Other quality filtering was performed with following qualifiers of PRINSEQ (lite v0.20.4) [11]: read length ≥ 50 nucleotides, mean quality score of read ≥25, proportion of ambiguous bases ≤1%, filter all kinds of duplicates, DUST score measuring low complexity ≤7. Quality filtering was confirmed with FastQC (v0.11.8; https://www.bioinformatics.babraham.ac.uk/projects/fastqc; 10.5.2021). Quality reads were aligned with STAR (v2.7.1a) [12] against human reference genome (GCF_000001405.26_GRCh38_genomic.fna from NCBI).

### Deconvolution analysis

Deconvolution analysis of different immune cell types was done utilizing a digital cytometry tool CIBERSORTx [7]. CIBERSORTx estimates the abundances of cell types in a mixed cell population, based on gene expression data and known connections between genes and cell types. CIBERSORTx provides an empirical *p*-value to evaluate deconvolution performance. The p-value is calculated by comparing the resulting cell type fractions with fractions that would have been obtained by random chance [13]. We ran CIBERSORTx utilizing the default LM22 dataset, consisting of 22 functionally defined human hematopoietic subsets [14]. In the dataset, these cell types are strictly named as: B cells naive, B cells memory, Plasma cells, T cells CD8, T cells CD4 nAaive, T cells CD4 memory resting, T cells CD4 memory activated, T cells follicular helper, T cells regulatory Tregs, T cells gamma delta, NK cells resting, NK cells activated, Monocytes, Macrophages M0, Macrophages M1, Macrophages M2, Dendritic cells resting, Dendritic cells activated, Mast cells resting, Mast cells activated, Eosinophils, and Neutrophils. In figures and tables we use this strict naming, but in the text we refer to cell types in more flexible manner. CIBERSORTx determines the proportions of these 22 cell subtypes using a signature matrix that consists of the expression levels of 547 signature genes. CIBERSORTx utilizes machine learning in the form of support vector regression to determine cell proportions. Further detail on the composition of the LM22 dataset can be found in the original CIBERSORT publication [15]. Other CIBERSORTx parameters were as follows: Batch correction was enabled, and the number of permutations set to 1000 for significance analysis. TPM normalized gene expression values, from region-specific brain samples taken from the studied 55 individuals, were used as the mixture matrix.

## Results

### Composition of the immune cells in the brain

**Fig. 1:**
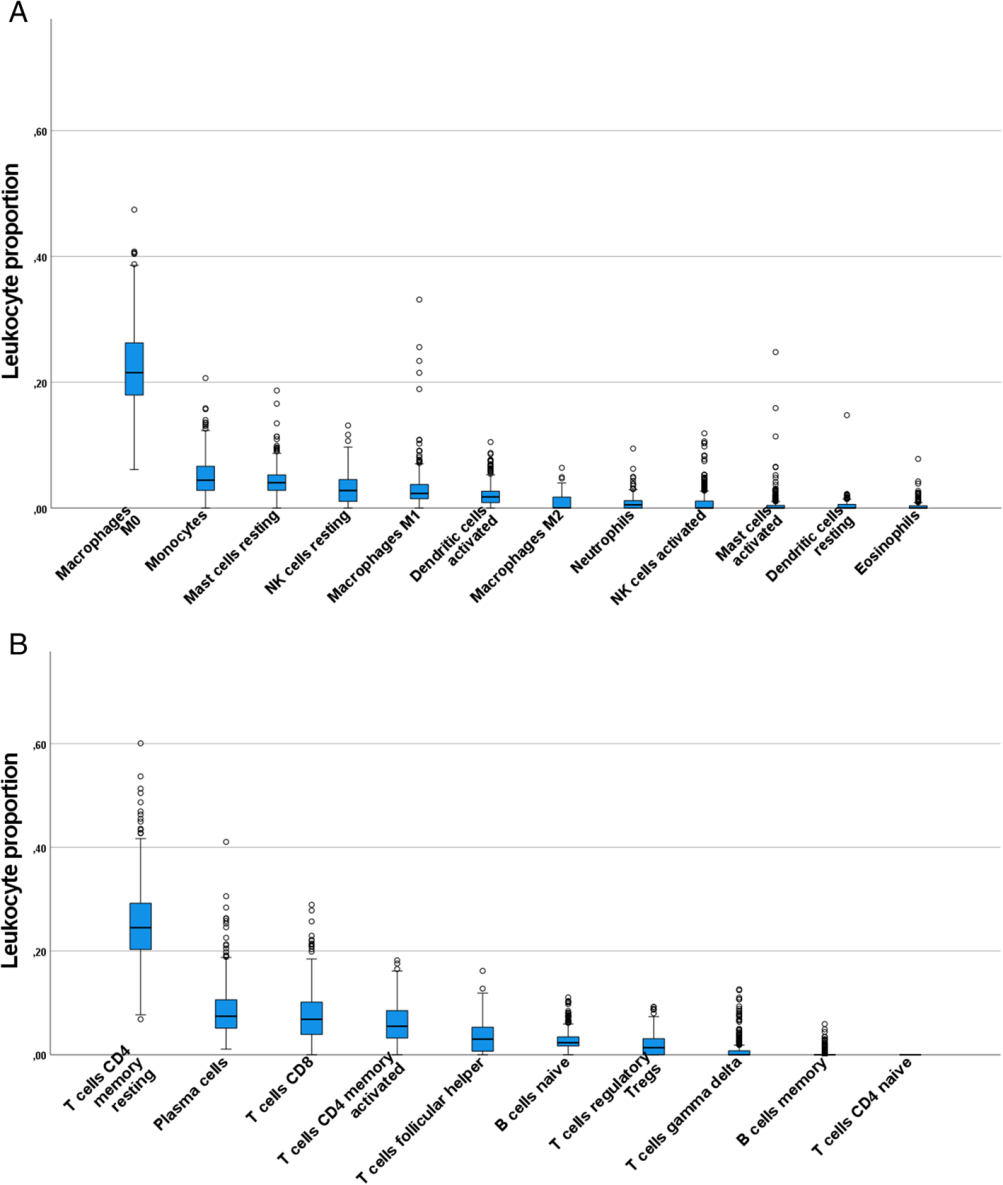
Distributions of leukocyte proportions in the CNS. Panel A shows proportions of innate immune cells and panel B proportions of adaptive immune cells. Number of samples is 378, with all brain regions combined

**Fig. 2:**
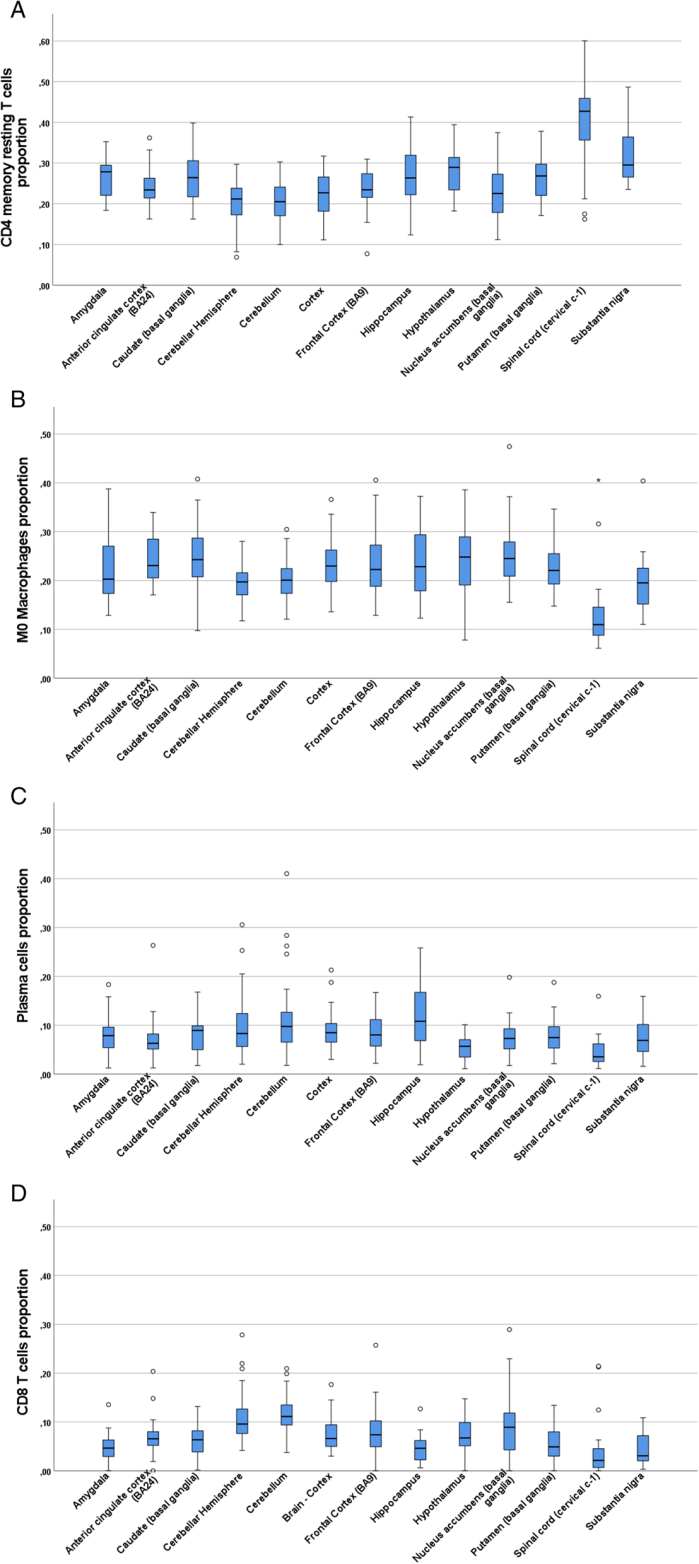
Distributions of the most abundant leukocytes analyzed separately for each brain region. Panel A: CD4 memory resting T cells, Panel B: M0 Macrophages, Panel C: plasma cells and panel D: CD8 T cells. Sample size varied for each brain region: amygdala (*n* = 24), anterior cingulate cortex (*n* = 24), caudate (*n* = 35), cerebellar hemisphere (*n* = 37), cerebellum (*n* = 39), cortex (*n* = 31), frontal cortex (*n* = 31), hippocampus (*n* = 28), hypothalamus (*n* = 25), nucleus accumbens (*n* = 34), putamen (*n* = 30), spinal cord (*n* = 20) and substantia nigra (*n* = 20)

To obtain an overall view of the infiltrating immune cells in the brain, the cell type distributions were first examined by omitting the fact that samples originate from different brain regions. The data in Fig. 1. shows the quantified distributions of the infiltrated immune cells in the brain separately in the case of innate immune cells and adaptive immune cells. The proportions of CD4 memory resting T cells and macrophages (M0) were clearly highest (median proportions 0.245 and 0.215, respectively). Additionally, plasma cells, CD8 T cells and CD4 memory activated T cells were present in moderate proportions; median proportion being higher than 0.05 (median proportions 0.074, 0.068, and 0.055, respectively). CD4 naïve T cells were completely absent in all samples. Variance in cell proportions is high, especially in the case of the most abundant cell types. Part of this variance seems to be explained by the slight differences in proportions between the different brain regions as shown in Fig. 2.

### Immune cell types in the different anatomic regions of the brain

The GTEx brain samples have been collected from 13 different anatomic regions (amygdala, anterior cingulate cortex, caudate basal ganglia, cerebellar hemisphere, cerebellum, cortex, frontal cortex, hippocampus, hypothalamus, nucleus accumbens, putamen basal ganglia, spinal cord and substantia nigra). It can be hypothesized that the anatomic location or microenvironment in the CNS would have an influence on the relative abundance and interactions of different immune cells. To assess this, the relative proportions of the most abundant cell types were examined brain region – vise.

Figure 2. shows the relative proportions of the four most abundant cell types (CD4 resting memory T cells, M0 macrophages, plasma cells and CD8 T cells) at different brain regions. It can be concluded that these cell types appear at different brain regions with relatively constant proportions. Sole exception was spinal cord, where proportions of CD4 memory resting cells were higher and proportions of M0 macrophages were lower than elsewhere in the brain.

### Aging-associated differences in the cell type proportions

Aging-effects associated with different immune cell proportions were examined by correlating the age of the individual with each cell proportion separately for each brain region. Tables 1 and 2 show all observed significant correlations for innate immune cells and adaptive immune cells, respectively. In the case of the innate immune system, significant age-correlations with cell types were mainly positive, whereas in the case of the adaptive immune cells significant correlations were negative in most cases.

**Table 1:**
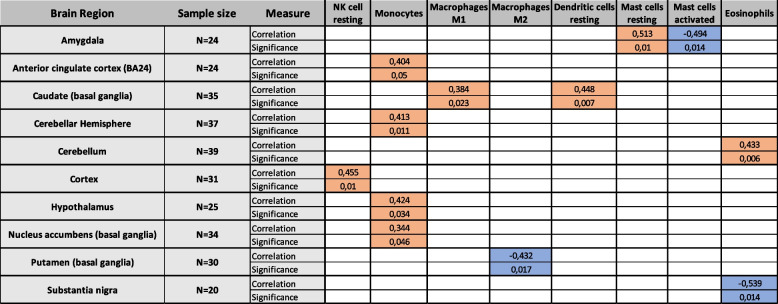
Brain region – vise aging-associated effects of the immune cell proportions of innate immune cells. Significant positive correlations are highlighted in orange and significant negative correlations are highlighted in blue. Table contains only those cell types and those brain regions where at least one significant correlation was observed. Correlations that were not statistically significant were omitted for better readability. Correlation was obtained with Spearman’s rank correlation and *p*-values less than or equal to 0.05 were considered significant

**Table 2:**
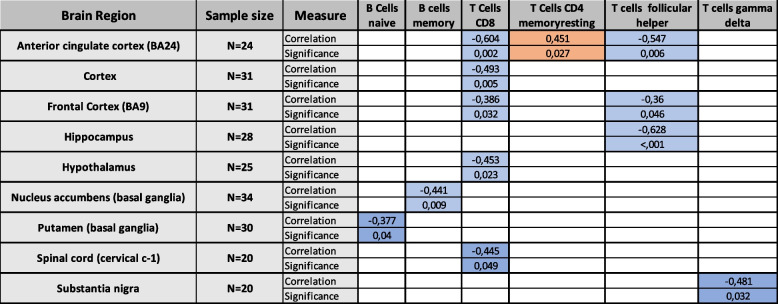
Brain region – vise aging-associated effects of the immune cell proportions of adaptive immune cells. Significant positive correlations are highlighted in orange and significant negative correlations are highlighted in blue. Table contains only those cell types and those brain regions where at least one significant correlation was observed. Correlations that were not statistically significant were omitted for better readability. Correlation was obtained with Spearman’s rank correlation and p-values less than or equal to 0.05 were considered significant

Several immune cell types had significant correlation with age at a single brain region. However, in the case of monocytes, CD8 T cells, and follicular helper T cells a consistent significant correlation to age could be seen at several brain regions. The proportion of monocytes correlated positively with age at anterior cingulate cortex (*r* = 0.404, *p* = 0.05), cerebellar hemisphere (*r* = 0.413, *p* = 0.011), hypothalamus (*r* = 0.424, *p* = 0.034), and nucleus accumbens (*r* = 0.344, *p* = 0.046). The proportions of CD8 T cells correlated negatively with age at anterior cingulate cortex (*r* = − 0.604, *p* = 0.002), cortex (*r* = − 0.493, *p* = 0.005), frontal cortex (*r* = − 0.386, *p* = 0.032), hypothalamus (*r* = − 0.453, *p* = 0.023), and spinal cord (*r* = − 0.445, *p* = 0.049). Finally, the proportions of follicular helper T cells correlated negatively with age at anterior cingulate cortex (*r* = − 0.547, *p* = 0.006), frontal cortex (*r* = − 0.36, *p* = 0.046), and hippocampus (*r* = − 0.626, *p* < 0.001).

## Discussion

It is now well established, that cells of the immune system infiltrate to CNS. However, implications of this are poorly understood. To shed some light into this, we analyzed the infiltrating immune cell proportions at different brain regions to investigate whether infiltrating mechanisms differ. Additionally, as aging is a relevant factor in the neurodegenerative diseases as well as in the functioning of the immune system, we analyzed whether age has effect on the immune cell infiltration at different brain areas.

The leukocyte types that were observed to be present in the whole brain in the highest proportions (representing a proportion of 0.05 i.e. 5% or more) were CD4 memory resting T cells, M0 macrophages, plasma cells, CD8 T cells, and CD4 memory activated T cells as shown in fig. 1.

The high level of CD4 T cells observed here could be related to their enhanced immune activity or to their enhanced infiltration capacity from blood. The immunological memory in the CD4 population is complex, involving several subtypes of memory cells (reviewed in [16, 17]). The resting memory cells probably represent cells in the quiescent, or resting, state and are therefore long-lived. Since they are long-lived, it is also possible that they are simply accumulating in the brain over time, resulting in their high proportion.

The most common macrophage type observed in the brain samples was M0. Macrophages M0 represent the unactivated macrophages that have potential to differentiate to pro-inflammatory M1 macrophages or anti-inflammatory M2 macrophages [18]. As macrophages play critical role in the tissue homeostasis, the high levels of the M0 macrophages in the brain could reflect this need. It is worth noting that CNS in also inhabited by the resident macrophages, the microglias, and the quantification of macrophages could be affected by the presence of microglia. However, the makers of CIBERSORT have attempted to address this potential issue of confounding cell types when creating the LM22 dataset [15]. The signature genes that the LM22 dataset relies on have been filtered with the aim to only match these specific leukocytes [15]. The LM22 dataset has also already been used to estimate infiltrating cell proportions in the brain [19]. Of the differentiated macrophages, the proportions of M1 macrophages were somewhat higher than that of M2 macrophages. It is worth pointing out that macrophage nomenclature has been a debated topic recently and the nomenclature is used inconsistently in literature [20]. In this work we have used the nomenclature that CIBERSORTx LM22 dataset uses (M0, M1, M2) [15].

The proportions of plasma cells and other cell types of the B cell lineage were relatively high in the studied brain samples. Plasma cells and other cell types of the B cell lineage are involved in the pathogenesis and physiology of the CNS and they can be found in the CNS of healthy individuals at least in small numbers [3]. It has been reported [2] that the plasma cell levels are increased in MS patients, suggesting that they are involved in the pathogenesis of this disease.

CD8 T cells have often been found in the CNS, e.g. in MS patients [2] or in CNS infections [4]. In these conditions the pathogenic mechanisms are mediated by a subset having the tissue-resident CD8 characteristics. The 22 infiltrating leukocyte subsets quantified in this work did not contain CD8 subsets.

When leukocyte proportions were analyzed without brain region separation, the observed variance was notably high. When infiltrating leukocyte proportions were studied separately for each brain region, the proportions of individual cell type were observed to be relatively homogenous between the 13 brain regions, with the exception of spinal cord as shown in fig. 2. The variation seen in fig. 1 is partly explained by these observations. The distinct proportions observed in the spinal cord could be due to simple anatomical differences. The homogeneity between the rest of the brain regions could imply that the infiltrating cells passively diffuse into the brain without any preference for the location.

Neutrophils are the most numerous leukocyte type in the blood, with a normal proportion of 40 to 70% of leukocytes [21], and yet are barely present in the studied brain samples at median proportion of 0.5%. This lower proportion in the brain could be related to the short lives of neutrophils. This also supports the idea that infiltrating cells in the CNS could be mainly long-lived cells that accumulate there.

As aging is associated with immune system changes [22] and many aging-associated neurodegenerative diseases have immunological pathophysiology [23], we analyzed whether age associates with quantified leukocyte proportions in different brain regions. Three leukocyte types (monocytes, CD8 T cells, and follicular helper T cells) were observed to correlate significantly with age on several brain regions as shown in tables 1 and 2. In the case of monocyte proportions, positive correlation with age was observed. Monocytes are known to contribute to age-related, systemic, low-level inflammation, which fits in what is known about inflammaging [19]. CD8 T cells are known to be affected by aging in terms of immunosenescence, which manifests as increased CD4/CD8 ratio mainly caused by decrease in CD8 T cell numbers in periphery [24]. Our data supports shows that this effect can also been seen in the CNS as we observed a negative correlation between CD8 T cell proportions and age. On the other hand, the accumulation of senescent CD8+/CD28- cells, that contribute to chronic inflammation is one hallmark of inflammaging [25]. CIBERSORTx-algorithm cannot differentiate between the subtypes of CD8 cells, but CD28 is one of the signature genes whose expression is taken into account in the determination of the cell proportions. Therefore, it is possible that samples from older individuals containing more senescent CD28- cells might have their CD8 proportions underestimated.

One limitation of this work is that though the GTEx derived data is from non-diseased tissue sites, the samples were gathered post-mortem. It is possible that this may affect the estimated cell type proportions. A future study utilizing fresher samples would have great value.

In summary, these data indicate that several types of immune cells are present in the CNS of healthy individuals. These cell types are known to be involved in the pathogenesis of several immune and inflammatory diseases of the CNS. It remains to be established whether these cells are early indicators of a pathogenic process or just signs of normal baseline immune activity. In addition, we observed aging-associated effect in proportions of monocytes, CD8 T cells, and follicular helper T cells, that are in line with what is known about inflammaging and immunosenescence.

## Data Availability

The raw RNA-seq data of the GTEx project analyzed in this work can be accessed for research purposes through the database of Genotypes and Phenotypes (dbGaP) system. The dbGaP accession number for the project is phs000424.v8.p2. Access to GTEx protected data, which includes the raw sequencing data, requires an approved dbGaP application.
